# Clinical Experience With an Aragonite-Based Scaffold Implant for Knee Cartilage Repair: A Multicenter Case Series

**DOI:** 10.7759/cureus.86127

**Published:** 2025-06-16

**Authors:** Nirav H Amin, Scott C Faucett, Charles Qin, Chaitu S Malempati, Ronak M Patel, Christopher P Dougherty, Arnold Lim, Corey Kendall, R. Kyle Martin, Cassandra A Lee

**Affiliations:** 1 Orthopaedic Surgery - Sports Medicine, Premier Orthopaedic and Trauma Specialists, Pomona, USA; 2 Orthopaedic Surgery - Sports Medicine, Centers for Advanced Orthopaedics, Washington, D.C., USA; 3 Sports Medicine, Cheshire Medical Center, Keene, USA; 4 Sports Medicine and Orthopedics, Med Center Health Orthopaedics and Sports Medicine, Bowling Green, USA; 5 Orthopaedic Surgery - Sports Medicine, Illinois Bone and Joint Institute, Westmont, USA; 6 Sports Medicine, Arkansas Center for Arthroscopy, Bentonville, USA; 7 Sports Medicine, Tenaya Medical Group, Merced, USA; 8 Sports Medicine, OrthoIndy, Indianapolis, USA; 9 Orthopedic Surgery, University of Minnesota, Minneapolis, USA; 10 Orthopaedic Surgery, University of California Davis, Sacramento, USA

**Keywords:** aragonite, cartilage, case series, chondral, data, knee lesions repair, osteochondral, safety, sports medicine

## Abstract

Background

There is a growing interest in the use of biomaterials to treat chondral and osteochondral knee lesions, given their ability to replicate the biological and functional properties needed for simultaneous cartilage and bone regeneration. A novel aragonite-based, cell-free biomimetic scaffold (CARTIHEAL AGILI-C^TM^, Smith + Nephew, UK) was developed for treating chondral and osteochondral defects in traumatic and osteoarthritic joints. A short-term follow-up study was designed to assess the safety and feasibility of this scaffold.

Materials and methods

This retrospective review included data from nine centers in the United States (US) between August 22, 2023 and December 30, 2024. Adult patients (≥18 years of age) who received the aragonite-based scaffold as standard of care for the treatment of knee chondral/osteochondral lesions in accordance with the manufacturer’s instructions for use were eligible. There were no prespecified exclusion criteria. All patients underwent magnetic resonance imaging (MRI) for radiologic assessment of knee cartilage lesions, which informed the development of presurgical plans. A diagnostic arthroscopy was performed before arthrotomy to confirm the radiographic findings obtained for preoperative planning. The primary endpoint was the incidence of early clinical and radiographic complications occurring within at least 30 days after the operation. Secondary endpoints included an assessment of the accuracy of presurgical planning relative to intraoperative findings, proportion of implants determined to be improperly implanted based on the first postoperative X-rays, change in the numeric pain rating score from baseline, and the proportion of patients cleared for various postoperative activities.

Results

A total of 33 patients (34 knees; mean age, 47.2 years; 18 (52.9%) male) were included. After a mean postoperative follow-up of 45.7 days (standard deviation, 14.4), one patient (2.9%) experienced a postoperative complication (pain, with no associated infection). Success rate was 96.97% (95% CI, 84.24-99.92). In 27 (79.4%) cases, the presurgical plan based on MRI was modified following arthroscopic visualization of the knee joint surface. Postoperative radiography revealed no complications for the 28 patients with data. Mean postoperative numeric pain rating significantly improved from 6.6 at baseline to 3.9 at follow-up (p<0.05). The majority of patients (n=24; 70.6%) were cleared for partial or full weightbearing by the 30-day postoperative follow-up visit.

Conclusions

This case series across multiple centers in the US demonstrates the clinical safety and feasibility of aragonite scaffold implantation. The flexibility of the scaffold in accommodating intraoperative findings and the low rate of early complications are encouraging.

## Introduction

Chondral and osteochondral knee lesions are distinct entities that commonly present in clinical practice. Chondral lesions result from cartilage degradation due to metabolic, genetic, or vascular factors, as well as acute trauma. Osteochondral lesions involve damage to both the articular cartilage and subchondral bone, typically due to trauma [1]. Studies of patients undergoing knee arthroscopy estimate the prevalence of chondral and osteochondral lesions at approximately 60% [2,3]. Their presence is associated with pain, functional impairment, and progression to osteoarthritis [3-5].

The historical surgical treatments for these lesions, such as arthroscopic debridement and microfracture, may provide short- to mid-term symptom relief but do not restore cartilage integrity, leading to a risk of long-term treatment failure [6-8]. Next-generation cartilage repair options, such as osteochondral allograft, osteochondral autograft, and matrix-based autologous chondrocyte implantation, may require two-stage procedures, have feasibility constraints, and/or limited ability to address the underlying osteochondral bone defect [9,10]. These shortcomings have driven the development of biomaterials designed to replicate the biological and functional properties needed for simultaneous cartilage and bone regeneration [9].

One promising biomaterial that achieves this goal is aragonite, a natural calcium carbonate derived from purified coral exoskeleton, which has a microarchitecture similar to human cancellous bone [11,12]. A novel aragonite-based, cell-free biomimetic scaffold (CARTIHEAL AGILI-C^TM^, Smith + Nephew, UK) was developed for treating chondral and osteochondral defects in traumatic and osteoarthritic joints. Its three-dimensional structure is designed to promote articular cartilage regeneration, facilitate adhesion and differentiation of bone marrow-derived stem cells into chondrocytes, promote chondrocyte proliferation and migration from native tissue, and support extracellular matrix deposition [10].

Preclinical studies have demonstrated the scaffold’s osteoinductive, osteoconductive, osteotransductive, and chondrogenic capabilities, supporting cartilage and subchondral bone restoration [12-18]. Clinically, the scaffold offers an off-the-shelf solution that can be implanted in a single-stage procedure. Several case series have demonstrated its safety and efficacy, with significant clinical improvements in patient-reported outcome measures and radiographic defect fill sustained for up to five years postoperatively [19-24]. These findings were validated in a randomized controlled trial (RCT) of patients with up to three high-grade cartilage defects (International Cartilage Regeneration & Joint Preservation Society ≥ grade 3a [25]), including those with mild-to-moderate osteoarthritis [24]. At a two-year follow-up, the scaffold demonstrated statistical superiority over debridement/microfracture in both the primary endpoint (Knee Injury and Osteoarthritis Outcome Score improvement [26]) and all secondary endpoints [24]. Further analysis at a four-year follow-up confirmed consistent efficacy regardless of lesion location, and that superior outcomes are seen with both men and women [27,28].

Although RCTs remain the gold standard for clinical evidence, their strict inclusion criteria and controlled conditions may not fully reflect real-world patient populations and surgical practices. As a result, there is growing interest in real-world evidence, which draws upon data collected from routine clinical experiences [29,30]. Advances in electronic health records have facilitated the collection of reliable real-world evidence [31], which is now considered an essential supplement to RCT data [29].

The purpose of the current study was to supplement existing clinical data on the aragonite-based scaffold by providing post-marketing evidence from multiple centers in which it was used for the treatment of chondral/osteochondral knee lesions. The primary endpoint was to evaluate the incidence of early clinical and radiographic complications occurring within at least 30 days after the operation. A secondary endpoint was to assess the accuracy of presurgical planning relative to intraoperative findings.

## Materials and methods

Study design 

This study was a retrospective review of data obtained from nine centers in the United States between August 22, 2023 and December 30, 2024. Adult patients (≥18 years of age) who received the aragonite-based scaffold as standard of care for the treatment of knee chondral/osteochondral lesions in accordance with the manufacturer’s instructions for use were eligible for inclusion. There were no prespecified exclusion criteria. Eligible centers were required to enroll at least two patients and should not have participated in prior clinical trials with this scaffold. 

This study was performed in line with the principles of the Declaration of Helsinki. The Western Institutional Review Board -Copernicus Group served as the central institutional review board (IRB) of record (approval number 20245232, received on January 3, 2025). All participating centers also applied for and received individual IRB approval. Due to the retrospective nature of the study, the requirement for individual patient consent was waived.

Patients were selected for surgery following a comprehensive review of symptoms, demographics, severity of osteoarthritis, and treatment goals. The surgical treatment was selected based on the surgeon's professional opinion, factoring in patient considerations when appropriate. Race and ethnicity were collected as part of routine care at study centers and were drawn from the electronic health records. All patients underwent magnetic resonance imaging (MRI) for radiological assessment of knee cartilage lesions, which informed the development of presurgical plans.

Surgical technique

The surgical technique used for implanting the study scaffold was as follows. With the patient under anesthesia, a diagnostic arthroscopy was performed to confirm the radiographic findings from preoperative planning, including the chondral lesion sizes and locations, and any associated pathology, such as meniscal damage, was treated accordingly. The defect site was accessed via an arthrotomy. A mini arthrotomy could be performed, but the exposure must allow for a perpendicular approach to the defect. A perpendicular aligner was used to visually assess the surface area of the placement of the implant. A dedicated instrument set was used to sequentially drill through the articular surface into the subchondral bone. The lesion was contained, meaning the drilled hole was completely within the cortical bone and a rim of cartilage. On the chondral surface, the prepared defect was abutting the edge of the chondral defect so that the prepared lesion touched the normal adjacent articular cartilage. Using the aligner, the guide pin was introduced perpendicular to the articular surface to the level of the laser line. A cannulated drill was used to remove the bone in a calibrated manner. A depth reamer was then hand-articulated clockwise until the laser line was buried 360° into the surrounding cartilage to ensure the full and correct depth of the hole was achieved. The shaper was used to obtain the conical shape of the socket and ensure clean preparation of the hole. The guide pin was then removed. A cartilage cutter was placed conically to cut the chondral surface and ensure smooth edges for the implant delivery. The defect was then thoroughly irrigated to ensure removal of any loose bone or cartilage tissue.

The implant was then introduced into the defect, hole side up, under digital pressure. The silicone end of the tamper was used to seat the implant into the prepared bed. The implant should be seated at least 2 mm below the articular surface. Depending on the characteristics and/or location of the lesion(s), the surgeon could choose to use multiple implants to effectively treat a single lesion. When using multiple implants for a single lesion, the location of the implants was triangulated to ensure that a bone bridge of at least 5 mm was preserved between the edges of the two adjacent implants, and that each implant touched as much healthy articular cartilage tissue as possible. The surgeons ensured that the implant was correctly placed before advancing to subsequent implants. 

Rehabilitation protocol

The participating surgeons prescribed specific rehabilitation protocols for each patient, considering their standard clinical practice, operative considerations including concomitant surgical procedures, and patient needs. Compared to other chondral treatments, the implant evaluated in the current study bears no weight after implantation, therefore, rehabilitation was dictated by patients’ symptoms and concomitant procedures performed. When implanted as an isolated procedure, the rehabilitation guideline used by the participating surgeons differed slightly for trochlear vs condylar lesions due to the mechanics and weightbearing nature of the compartments. For trochlear lesions, the rehabilitation guideline for most patients in the immediate postoperative period (zero to six weeks) was brace-locked extension for weight bearing as tolerated, with progression to full weight bearing in six weeks. No restriction of range of motion (ROM) was required, and progress as tolerated to full ROM by week six to eight was observed. For the condyle lesions, in the immediate postoperative period, no restrictions of ROM were required, with full ROM typically achieved by sixth week. Weight bearing was restricted for the first two to three weeks, with progression to full weight bearing observed by weeks four to six. Following the immediate postoperative period, no bracing or restrictions on ROM or weight bearing were required. For all lesions, a regular exercise bike was recommended as early as possible, with increased resistance at approximately six weeks. 

Outcomes

A retrospective chart review was conducted by trained study staff at each center. To ensure patient privacy and data consistency, de-identified information was entered into a standardized case report form. The aggregated data from all study centers were analyzed collectively by a statistician not affiliated with any of the centers. 

Analysis included demographic information (age, sex, body mass index, clinical history, prior cartilage repair procedures), preoperative assessment (MRI findings to support treatment planning), and intraoperative data (lesion characteristics, implant numbers, and changes to the presurgical plan). 

Outcomes occurring within at least 30 days of the index surgery were analyzed. The primary outcome was the proportion of patients who experienced postoperative complications at approximately 30 days post-surgery. Complications were defined as one or more of the following: surgical site infections, including septicemia or deep infections in the operated joint; wound disruption or wound complication, including wound dehiscence, hematoma, site drainage, or superficial infection; hospital admission related to device and/or procedure; secondary procedure (i.e., injection) related to the device and/or procedure; pulmonary embolism; deep vein thrombosis; allergic reaction; all-cause reoperation; device removal; or other (i.e., bone marrow edema, bone cyst, osteophyte formation).

Secondary outcomes were as follows: (1) proportion of implants determined to be improperly implanted based on the first postoperative X-rays, with improper implantation defined as an instance of one or more of the following radiographic findings for each implant: implant cracked and/or broken, implant not recessed at least 2 mm with respect to the articular surface (implant “proud”), or entrapment of soft tissue between implants (implant impingement); (2) proportion of patients where the surgical plan determined by the surgeon based on presurgical MRI assessment was changed intraoperatively, specifically with respect to the number of treatable lesions, lesion size (within 0.5 cm^2^), lesion location(s) (trochlea, condyle), and lesion type (chondral vs subchondral lesions); (3) change from baseline in the numeric pain rating score; and (4) proportion of patients cleared for the following activities: partial weight bearing, full weight bearing, unassisted walking (defined as walking without mobility aids for short distances and to perform basic daily activities), light activities like standing or walking for up to two hours/day and frequently carrying items weighing up to 10 pounds, moderate activities like standing or walking for up to four hours/day and frequently carrying items weighing items up to 25 pounds, and resuming normal daily activities (i.e., return to baseline activity level or higher).

Statistical analysis

This study had an anticipated inclusion of approximately 45 patients/surgeries, with no statistical power analysis.

Descriptive statistics were used to summarize patient characteristics, surgical details, and outcomes. Continuous variables were reported as means with standard deviations (SD), whereas categorical variables were presented as frequencies and percentages. Where appropriate 95% confidence intervals (CI) were calculated using Binomial Clopper Pearson analysis. Data were analyzed using SAS Version 9.4 or later (SAS Institute Inc., Cary, NC). 

## Results

Thirty-three patients and 34 knees were included in the study (Tables 1, 2), with one patient undergoing bilateral implantation.

**Table 1:** Demographic characteristics BMI, body mass index; SD, standard deviation. Data are expressed as number (%) unless otherwise noted.

Characteristic	Value
Patient age, mean (SD), years, n=34	47.2 (12.5)
Sex, n=34	
Male	18 (52.9)
Female	16 (47.1)
Race, n=22	
Black or African-American participants	2 (9.1)
White participants	17 (77.3)
Other	3 (13.6)
Ethnicity, n=16	
Hispanic/Latino participants	4 (25.0)
Non-Hispanic/Latino participants	12 (75.0)
BMI, mean (SD), n=24	31.2 (6.2)
Regular daily activity level, n=17	
Sedentary	3 (17.6)
Light	6 (35.3)
Moderate	6 (35.3)
Heavy	2 (11.8)
Smoking status, n=24	
Current smoker	2 (8.3)
Former smoker	1 (4.2)
Never smoker	21 (87.5)

**Table 2:** Baseline patient characteristics COPD, chronic obstructive pulmonary disease; MACI, matrix-induced autologous chondrocyte implantation; OATS, osteochondral autograft transfer; OCA, osteochondral allograft.

Characteristic	Value
Index knee, n=34	
Left	15 (44.1)
Right	19 (55.9)
Osteoarthritis severity (Kellgren-Lawrence grade), n=20	
0	10 (50.0)
1	4 (20.0)
2	4 (20.0)
3	2 (10.0)
Duration of symptoms prior to surgery, n=22	
<6 months	7 (31.8)
6-12 months	6 (27.3)
13-24 months	6 (27.3)
>24 months	3 (13.6)
Conditions impacting healing	
Diabetes mellitus, n=31	6 (19.4)
Systemic steroid use, n=31	0 (0)
Hypertension, n=32	4 (12.5)
COPD/Dyspnea, n=27	1 (3.7)
Bleeding disorder, n=34	0 (0)
Knee treatments in last 12 months	
Any treatment, n=34	18 (52.9)
Injections in the index knee, n=29	12 (41.4)
Opiate prescription for knee pain, n=28	3 (10.7)
Microfracture, n=30	1 (3.3)
Cartilage debridement, n=31	4 (12.9)
Coblation, n=30	0 (0)
MACI, n=31	0 (0)
OCA, n=31	0 (0)
OATS, n=31	0 (0)
Other non-surgical treatment, n=28	12 (42.9)
Patient-reported knee signs/symptoms	
Catching or locking, n=21	13 (61.9)
Popping or clicking, n=21	17 (81.0)
Instability, n=22	8 (36.4)
Swelling, n=24	21 (87.5)
Stiffness, n=21	16 (76.2)
Reduced range of motion, n=20	11 (55.0)

Forty lesions were identified on preoperative MRI, conducted a mean of 65.7 (SD, 46.6; min=8, max=149) days before the surgery. Last preoperative visit preceded the surgery by a mean of 28.5 (SD, 24.4; min=1, max=85) days. Intraoperatively, 48 lesions were identified. Lesion characteristics determined preoperatively on MRI and intraoperatively are presented in Tables 3, 4. 

**Table 3:** Preoperative and intraoperative patient-level lesion characteristics SD, standard deviation. Data are expressed as number (%) unless otherwise noted.

	Preoperative value	Intraoperative value
Number of lesions per patient	n=31	n=34
Mean (SD)	1.3 (0.5)	1.4 (0.6)
1	23 (74.2)	22 (64.7)
2	7 (22.6)	10 (29.4)
3	1 (3.2)	2 (5.9)
Cumulative lesion size per patient	n=20	n=34
Mean (SD), cm^2^	3.9 (2.6)	5.8 (5.7)
<4 cm^2^	11 (55.0)	17 (50.0)
≥4 cm^2^	9 (45.0)	17 (50.0)
Number of concomitant procedures		n = 34
0 procedures		11 (32.4)
1 procedure		10 (29.4)
>1 procedure		13 (38.2)

**Table 4:** Preoperative and intraoperative lesion-level lesion characteristics SD, standard deviation. Data are expressed as number (%) unless otherwise noted. *Multiple lesions per patient are possible.

	Preoperative value	Intraoperative value
Lesion location*	n=39	n=46
Trochlea	7 (17.9)	11 (23.9)
Medial femoral condyle	26 (66.7)	30 (65.2)
Lateral femoral condyle	6 (15.4)	5 (10.9)
Lesion size*	n=25	n=47
Mean (SD), cm^2^	3 (2.4)	4.2 (5.1)
<4 cm^2^	20 (80.0)	33 (70.2)
≥4 cm^2^	5 (20.0)	14 (29.8)
Lesion type	n=36	n=33
Chondral	19 (52.8)	20 (60.6)
Osteochondral	17 (47.2)	13 (39.4)

**Figure 1:**
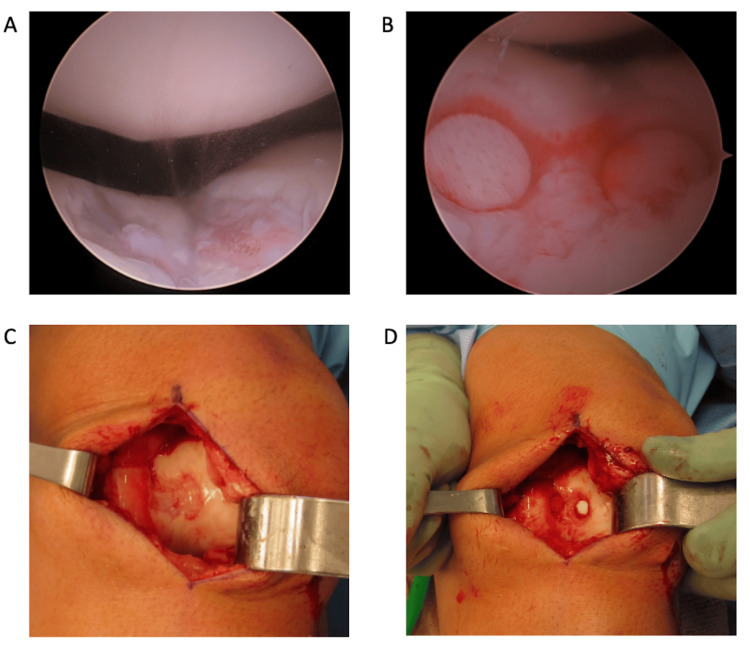
Intraoperative photographs of the cartilage defect (A,C) and the implanted aragonite scaffold (B,D) in the patient who received the scaffold bilaterally A,B: Surgical field of view during arthroscopic chondroplasty of the right knee. The defect, located in the central trochlea (21 mm × 17 mm in size) was repaired with two aragonite implants C,D: Surgical field of view during open chondroplasty of the left knee to repair the lesion (1 mm × 18 mm in size) with two aragonite implants. Aragonite implants: CARTIHEAL AGILI-C^TM^, Smith + Nephew, UK

Intraoperative photographs of the implant are provided in Figure 1.

Primary endpoint

Mean postoperative follow-up duration was 45.7 (SD, 14.4; min=14, max=81) days. On follow-up, one (2.9%) patient experienced a postoperative complication. The 47-year-old male patient presented to the emergency department on postoperative day three, with complaints of significant and uncontrolled pain in the index knee. His history included chronic opioid use prior to surgery but was otherwise unremarkable. He was readmitted for pain control. Diagnostic computed tomography and radiography were unremarkable for any device complications. Infection was ruled out with index knee aspiration, and the aspirate featured hemarthrosis. The patient was discharged after two days of hospitalization.

One patient (2.9%) reported increased stiffness in the index knee compared to baseline at the first postoperative follow-up. The patient was followed for suspected arthrofibrosis. The diagnosis was confirmed and treated with manipulation under anesthesia outside of the study window. 

Success rate based on clinical data from 32 of 33 patients with no complications was 96.97% (95% CI, 84.24-99.92).

Secondary endpoints

Postoperative radiography was performed a mean of 33 (SD, 36; n=18) days after the surgery to assess any postoperative complications, implant breakage, implant recession less than 2 mm from articular surface, implant impingement on or conversion with another implant, and other complications. It revealed no complications for the 28 (82.4%) subjects with data. Six (17.6%) patients did not have a postoperative X-ray within the study follow-up window. For two complication categories (any complications and implant breakage), data were available for 25 (73.5%) and missing for nine (26.5%) patients. All patients demonstrated successful implantation on postoperative radiographs. Success rate based on radiographic data from 25 of 25 patients with no negative radiographic findings was 100% (95% CI, 86.28-100). 

In nine (26.5%) cases, the presurgical plan based on imaging assessment was executed as intended. However, in 25 (73.5%) cases, the plan was modified following arthroscopic visualization of the knee joint surface. The diagnostic imaging report (MRI) was considered correct if the lesion number, type, location, and size identified corresponded to those of treated lesions from the operative note. For the cases where changes were made, modifications involved adjustments to the number and/or location of scaffolds. Between the preoperative MRI and intraoperative arthroscopic assessments, the number of treatable lesions was consistent in 27 (79.4%), lesion type in 21 (61.8%), and lesion location in 24 (70.6%) patients. In 12 (35.3%) cases, intraoperative modifications to the surgical plan were required because the imaging assessment did not provide complete information about the cartilage defect(s). In an additional 10 (29.4%) cases, lesion size measured on MRI differed from the intraoperative lesion size measurement by more than 5 mm. MRI data identified 41 total treatable lesions, while an additional seven treatable lesions were identified intraoperatively. 

After a mean follow-up of 47.5 days, mean postoperative numeric pain rating was 3.9 (SD, 2.6; n=16), which corresponds to an improvement from the mean preoperative pain rating of 6.6 (SD, 1.8; n=12). The preoperative pain rating was available for 12 patients, all of whom contributed to the postoperative rating. 

The proportion of patients cleared for activities on follow-up is presented in Table 5. 

**Table 5:** Postoperative findings (mean follow-up of 47.5 days) SD, standard deviation. ^*^Unassisted walking was defined as walking without mobility aids on even ground for short distances and to perform basic daily activities.

	Value
30-day opioid use, n=22	2 (9.1)
Current use of assistive walking devices (crutches), n=23	12 (52.2)
Surgical clearance for unassisted walking*, n=27	14 (51.9)
Weight bearing status, n=33	
Full weight bearing	12 (36.4)
Partial weight bearing	12 (36.4)
Non-weight bearing	9 (27.3)
Maximum active range of motion, mean (SD), degrees, n=30	108.9 (27.8)
Numeric pain rating score, mean (SD), n=16	3.9 (2.6)
Highest level of activity for which patient is surgically cleared, n=24	
Sedentary	10 (41.7)
Light	11 (45.8)
Moderate	3 (12.5)

The majority of patients (n=24; 70.6%) were cleared for partial or full weight bearing by the 30-day post-operative follow-up visit. Of the nine (26.5%) patients who were non-weight bearing, seven had concomitant meniscal procedures which required a more conservative rehabilitation protocol. 

## Discussion

The most important finding of this multicenter case series, the first to provide post-marketing evidence for this device from a real-world setting, is that this aragonite-based scaffold has a favorable safety profile, with a low 30-day complication rate. 

Complications in the early postoperative period are an outcome of interest for this novel device due to its mode of action. The porous architecture of the scaffold supports the influx and differentiation of bone marrow-derived stem cells into chondrocytes and extracellular matrix deposition [10]. Particular diligence is required in employing an aseptic technique during implantation, including an additional change of gloves, to prevent infection-related complications, which this study was designed to assess. The only postoperative complication in this study was related to pain control, with infection being ruled out. This supports the favorable safety profile of this scaffold, which is in line with the complication rate at 30 days for various isolated knee cartilage restoration procedures reported by Bohlen et al. [32].

Notably, this study provides the first documented case of bilateral aragonite implantation, which was completed without complications. Bilateral MRI demonstrated a trochlear chondral defect in each knee. The patient strongly desired a single-step procedure and accepted a recommendation of the aragonite scaffold. The patient’s postoperative course was uncomplicated, and he achieved weight bearing and returned to modified work duty six weeks after the surgery. 

After satisfaction with recovery from the right knee surgery, the patient elected to receive chondroplasty and aragonite implantation on the left side, to be performed with a minimal delay to limit overall downtime. The surgery on the left knee was performed two months after the right knee. At five weeks postoperatively, at the time of this writing, the patient reported no pain while walking and only mild discomfort with stairs. 

In over 75% of the cases, the presurgical plan based on MRI assessment changed during surgery, primarily due to discrepancies in lesion grade, lesion size, and cartilage quality compared to the MRI findings. Such discrepancies highlight the importance of performing an arthroscopic assessment in cartilage repair procedures. They also exemplify the versatility of this implant in adapting to changing surgical requirements discovered intraoperatively. The off-the-shelf nature of the scaffold, as well as the single-step surgical procedure used, allowed surgeons to adapt their plans intraoperatively to treat cartilage defects as identified, when identified. 

Improvement in pain on follow-up suggests that the procedure was successful and associated with favorable outcomes in the immediate postoperative period. Most patients achieved at least partial weight bearing on follow-up. This is in line with the recommended rehab protocol for this procedure, which indicates that most patients can begin at least partial weight-bearing immediately after the surgery and should achieve full weight-bearing six weeks after the operation. A previously published rehabilitation protocol states that patients who received the scaffold were expected to achieve full weight bearing between three and six weeks, but this may be largely dependent on patient-specific factors and concomitant procedures performed. Patients should be allowed to progress as tolerated based on the most conservative rehabilitation program, as no harm to the implant and/or recovery is expected with delayed weight bearing [24].

A key strength of this study is its multicenter design, which enhances the generalizability of the findings compared with single-center studies. Participating surgeons had limited experience with the scaffold implantation, not having enrolled patients in prior clinical studies of this device, ensuring that their results were reflective of the learning curve with this novel device. 

Limitations

The study was designed with a short follow-up period to focus primarily on early safety outcomes. Long-term studies are needed to assess the durability of the scaffold and its impact on patient-reported outcomes. 

The study employed a small sample size of 33 patients, which was below the anticipated inclusion of approximately 45 patients/surgeries. This contributes to the potential lack of generalizability to a larger population, as patients in the current study presented with several heterogenous comorbid knee conditions, the treatment approaches to which may not be reflective of broader use.

The lack of a comparison group limits the ability to draw conclusions about the relative efficacy of this scaffold compared to other cartilage repair techniques.

The data for the present study was retrospectively extracted from electronic health records. Due to variations in the standard of care and clinical documentation practices, some data were not collected or not recorded, resulting in a relatively high proportion of missing data. 

Possible variability in rehabilitation protocols between the sites may limit the interpretation of these findings. Previously published controlled studies established uniform rehabilitation protocol recommendations for this implant, and the current results supplement these data, providing insights into how this scaffold performs in everyday clinical practice, where variations in rehabilitation protocols are expected. Similarly, MRI characteristics and the protocol used are expected to vary between study sites.

Retrospective study designs have inherent limitations. To reduce potential bias, several measures were implemented. Consecutive enrollment helped mitigate potential for selection bias, whereas prespecified primary outcome measures and statistical analysis minimized reporting bias. Additionally, study centers were systematically selected to reduce bias.

## Conclusions

This case series demonstrates the short-term clinical safety and feasibility of aragonite scaffold implantation across multiple US centers after a mean follow-up of 45.7 days. The flexibility of the scaffold in accommodating intraoperative findings and the low rate of early complications are encouraging. These findings provide a foundation for further research and clinical application of this novel cartilage repair technology. Future studies should focus on long-term outcomes, patient-reported measures, and comparative effectiveness against established cartilage repair techniques.
